# Reliability of IMU-Derived Temporal Gait Parameters in Neurological Diseases

**DOI:** 10.3390/s22062304

**Published:** 2022-03-16

**Authors:** Clint Hansen, Christian Ortlieb, Robbin Romijnders, Elke Warmerdam, Julius Welzel, Johanna Geritz, Walter Maetzler

**Affiliations:** 1 Department of Neurology, University Hospital Schleswig-Holstein, Arnold-Heller-Strasse 3, Haus D, 24105 Kiel, Germany; c.hansen@neurologie.uni-kiel.de (C.H.); ct.ortlieb@outlook.de (C.O.); r.romijnders@neurologie.uni-kiel.de (R.R.); j.welzel@neurologie.uni-kiel.de (J.W.); j.geritz@neurologie.uni-kiel.de (J.G.); 2 Digital Signal Processing and System Theory, Institute of Electrical and Information Engineering, Kiel University, Kaiserstrasse 2, 24143 Kiel, Germany; 3 Division of Surgery, Saarland University, 66421 Homburg, Germany; elke.warmerdam@uni-saarland.de

**Keywords:** gait, walking, wearable sensors, reliability, neurology, inertial measurement units

## Abstract

Evaluating gait is part of every neurological movement disorder assessment. Generally, the physician assesses the patient based on their experience, but nowadays inertial measurement units (IMUs) are also often integrated in the assessment. Instrumented gait analysis has a longstanding tradition and temporal parameters are used to compare patient groups or trace disease progression over time. However, the day-to-day variability needs to be considered especially in specific patient cohorts. The aim of the study was to examine day-to-day variability of temporal gait parameters of two experimental conditions in a cohort of neurogeriatric patients using data extracted from a lower back-worn IMU. We recruited 49 participants (24 women (age: 78 years ± 6 years, BMI = 25.1 kg/m^2^ and 25 men (age: 77 years ± 6 years, BMI = 26.5 kg/m^2^)) from the neurogeriatric ward. Two gait distances (4 m and 20 m) were performed during the first session and repeated the following day. To evaluate reliability, the Intraclass Correlation Coefficient (ICC2,k) and minimal detectable change (MDC) were calculated for the number of steps, step time, stride time, stance time, swing time, double limb support time, double limb support time variability, stride time variability and stride time asymmetry. The temporal gait parameters showed poor to moderate reliability with mean ICC and mean MDC95% values of 0.57 ± 0.18 and 52% ± 53%, respectively. Overall, only four out of the nine computed temporal gait parameters showed high relative reliability and good absolute reliability values. The reliability increased with walking distance. When only investigating steady-state walking during the 20 m walking condition, the relative and absolute reliability improved again. The most reliable parameters were swing time, stride time, step time and stance time. Study results demonstrate that reliability is an important factor to consider when working with IMU derived gait parameters in specific patient cohorts. This advocates for a careful parameter selection as not all parameters seem to be suitable when assessing gait in neurogeriatric patients.

## 1. Introduction

While gait analysis is widespread, especially in clinical applications [1], sports rehabilitation [2] and equipment testing [3], it is most of the time restricted to laboratories and supervised assessments. Portable solutions such as inertial measurement units (IMUs) allow measurements outside of the laboratory in real world situations. IMUs usually contain accelerometers, gyroscopes and magnetometers, and the subsequent extraction of spatiotemporal gait parameters [4,5] is often based on sensor fusion algorithms [6,7]. While the validity of marker-based motion capture is rarely questioned [8], the reliability and validity of IMU-based motion capture is under constant evaluation [9] and the reliability of IMU-based gait parameters is rarely reported.

The ability to walk is an essential prerequisite to engage in everyday activities [10,11]. Changes in walking ability serve as risk factors for disabilities or worsening of health status and can lead to reduced quality of life, particularly when falls occur [12]. In order to assess reliability of gait parameters, long gait periods are favorable. Most studies focus, therefore, on the assessment of gait using instrumented treadmills to avoid the analyses of few isolated steps [13]. Since such equipment is not always available, and patients suffering from neurological diseases often show impaired walking behavior [14], this is not always possible [13]. Here, IMUs show a great advantage over stationary gait analysis systems: They can be used flexibly, and can also be used effectively for the evaluation of gait distances relevant to everyday life. These are often short, depending on what the affected person does in everyday life and what the structural situation is. It is therefore relevant to understand the validity, including the reliability, of IMUs in the context of recording such gait episodes, including relatively short gait episodes.

Reliability defines the extent to which a measurement is free from random error and is often separated into relative reliability and absolute reliability [15]. Relative reliability is assessed based on the intraclass correlation coefficient (ICC) indicating to what degree repeated measurements are consistent. Absolute reliability can be computed by the standard error of measurement (SEM) indicating the variability in scores upon repeated testing [16]. Based on the SEM, the minimal detectable change (MDC) can be computed which defines the smallest amount of change required to designate a change as real and beyond random error [17]. The systematic assessment of random errors that could affect the relative reliability of the parameters needs to be addressed. If a “significant” improvement is shown for multiple valid parameters but the differences are still within the MDC range, the results need to be interpreted with caution [18].

Therefore, the aim of this study is to assess the reliability of standardized gait assessment by establishing the MDC for a sample of temporal gait parameters in two different walking conditions (4 & 20 m walk) in a cohort of neurogeriatric patients. Neurogeriatric patients usually have marked gait limitations and thus seem ideally qualified for such a reliability study especially when using a cohort specific validated gait algorithm.

## 2. Materials and Methods

### 2.1. Participants

A total of 49 participants took part in this study [24 women (age: 78 years ± 6 years, BMI = 25.1 kg/m^2^) and 25 men (age: 77 years ± 7 years, BMI = 26.45 kg/m^2^)]. All participants were inpatients at the neurogeriatric ward of the Neurology Center at the University Hospital of Kiel between September 2017 and December 2019 where they volunteered to take part in the study ([19]). Among the variety of diagnoses, the three most common diagnoses (60% of all investigated diagnoses) were stroke (n = 16), Parkinson’s disease (n = 6) and back pain (3).

The participants were included when they were over 60 years, able to stand independently and walk three meters without personal assistance (walking aids allowed) as well as presence of at least one neurological disorder [19]. Participants were excluded when their fall risk was determined to be too high (>2 falls in the previous week), corrected visual acuity < 60%, ≤5 points in the Montreal Cognitive Assessment (MoCA) test [19,20], current or past chronic substance abuse (except nicotine) and not able to perform at least one of the walking tasks.

The study was approved by the ethics committee of the medical faculty of the University of Kiel (No. D427/17) and all participants provided written informed consent prior to participation.

### 2.2. Gait Assessment

Two standardized IMU-instrumented gait assessments were performed within 24 h on two consecutive days at the same time in the afternoon. The gait assessments always took place under the same conditions, in a defined test area (corridor of the 4th floor of the Neurocenter of UKSH, Campus Kiel, Kiel, Germany), with no external disturbances and with standardized instructions. The assessment included a 4 m and a 20 m walking task at preferred walking speed. Participants were asked to start walking once the examiner gave them a start sign and walked until the end of the respective walking distance, indicated by a colored tape on the corridor floor [19].

Each participant was equipped with a wearable IMU system (Rehawatch^®^, Hasomed, Magdeburg, Germany) worn at the lower back (L4-L5). The IMU contained a 3D accelerometer (±8 g), a 3D gyroscope (±2000°/s) and a 3D magnetometer (±1.3 Gs). The IMU system was securely fixed on the lower back using an elastic belt which was placed over the shirt of the participant.

### 2.3. Data Processing

The IMU data were processed with MATLAB (MathWorks, Nantick, MA, USA) using a validated step detection algorithm [21]. In addition to the 4 and 20 m walking task, we analyzed only the steady state walking phase from the 20 m task by removing the first three and last three steps [22].

The collected parameters provided information about the number of steps [n], step time [s], stride time [s], stance time [s], swing time [s], double limb support time [s], double limb support variability [s], step time asymmetry [s], step time variability [s] [21].

### 2.4. Statistical Analysis

The mean value (mean = M) and standard deviation (SD) for each of the two measurements were calculated.

Relative reliability. The relative reliability was expressed by the ICC type (2,k) [23,24]. The ICC is used to evaluate both systematic and random errors that could affect the relative reliability of the exercises. A 95% confidence interval (CI) was given for the ICCs. An ICC of >0.9 indicated excellent reliability, an ICC of 0.75–0.9 indicated good reliability, an ICC between 0.5–0.75 indicated moderate reliability, and an ICC < 0.5 indicated poor reliability [15,25].

Absolute reliability. The absolute reliability, which describes the within-participant variability due to repeated measurements, was determined by the $\mathrm{SEM}=\mathrm{SD}\sqrt{1-\mathrm{ICC}}$.

MDC represents the change that is not due to random variations of the measurements. MDC was calculated for a 95% confidence interval as MDC95=SEM × 1.96 × 2.

The z-value of 1.96 is that used for a normally distributed two-sided table with a 95% confidence interval, and 2 is used to account for the variance of the two measurements. The MDC95 is also expressed as a percentage, and defined as $\mathrm{MDC}\%=\frac{\mathrm{MDC}95}{\mathrm{mean}}\times 100$.

The mean value corresponds to the mean of the respective parameters for all measurements of the two assessments. MDC95% represents the minimum detectable change presented as a percentage that is not due to random variations of the measurements [15].

## 3. Results

### 3.1. 4 m Walk

For the 4 m walk, the participants required 8 ± 2 steps. Most of the gait parameters showed moderate reliability values. The number of steps (ICC = 0.67) reached the highest reliability values among the parameters examined. Stride time variability (ICC = 0.31) and stride asymmetry (ICC = 0.21) showed poor reliability values (Table 1).

The parameters, step and stride time, and swing and stance time were in the MDC range of 12–22%. These parameters are thus in a range in which the measured value must have changed by about an eighth to a fifth in order to reliably assume a relevant change. The number of steps and double support time were in an MDC range of 35–42%. These parameters are thus in a range in which the measured value must have changed by more than one third to reliably assume a relevant change. Stride time variability, double support time variability and asymmetry, showed MDC values of 100–188%. Thus, the measured value must have changed by more than double to reliably assume a relevant change.

### 3.2. 20 m Walk

For the 20 m walking distance, the participants required 35 ± 6 steps. Most of the gait parameters showed acceptable reliability values, whereby double limb support time variability (ICC = 0.34) and stride time variability (ICC = 0.19) showed poor reliability. The number of steps (ICC = 0.87) reached the highest reliability values among the parameters examined, which can be classified as good reliability (Table 2).

The parameters number of steps, step time, stride time, stance and swing time and double limb support time were in the MDC range of 12–17%. These parameters are thus in a range in which the measured value must have changed by one eighth to one sixth to reliably assume a relevant change. Double limb support time variability/asymmetry and stride time variability showed MDC values of 91–150%. This is in a range in which the measured value must have changed by more than double to reliably assume a relevant change.

### 3.3. 20 m Walk (Steady State)

For the steady state walking phase of the 20 m distance, the participants required an average of 29 ± 6 steps. Most of the gait parameters showed moderate to acceptable reliability values, and only double limb support time variability (ICC = 0.48) showed poor reliability. The number of steps (ICC = 0.87) reached the highest reliability values among the parameters examined. MDC values were comparable to those observed in the 20 m walk including acceleration and deceleration phase (Table 3).

### 3.4. MDC% Values of All Parameters and Experimental Conditions

**Figure 1:**
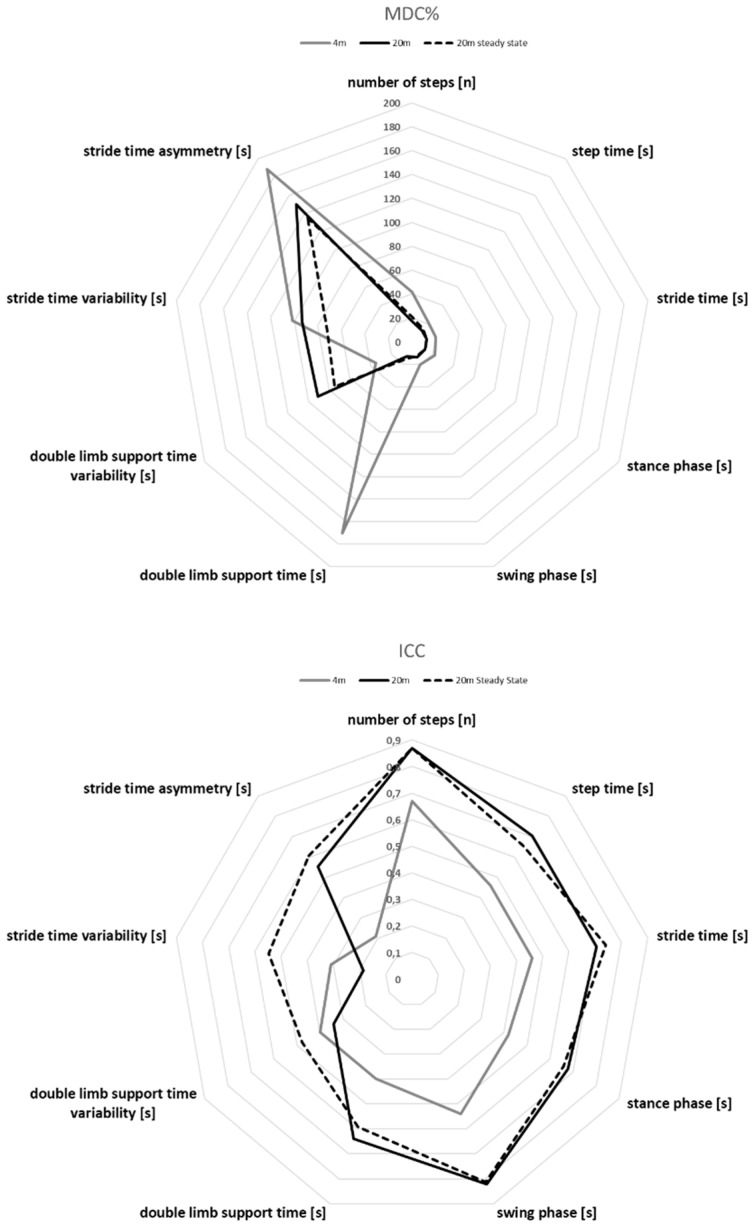
The ICC and MDC% values are shown for the nine parameters for the 4 m walking, 20 m walking distance and the 20 m walking (steady state), highlighting the reliability improvement with walking distance.

The MDC% values of all exercises and associated parameters are shown in Figure 1. The parameters step time, stride time, stance time and swing time showed MDC% values of 12–21% for all walking trials. They are followed by the number of steps with MDC% values of 17% to 42%. The highest MDC% values were calculated for the parameters of double limb support time, double limb support time variability, stride time variability and stride time asymmetry, with values up to 188%. The parameters of double limb support time variability, stride time variability and stride time asymmetry were always in a MDC% range of >70%, except for double limb support time variability in the 4 m walking condition with an MDC% of 35%. On a general note, the MDC% values decrease with walking distance. This shows especially when comparing the MDC% values of the 4 m and 20 m walking distance. The MDC% values decrease even further looking at steady-state walking even though the walking distance decreases compared to 20 m walking distance.

## 4. Discussion

The aim of this study was to evaluate the day-to-day variability of gait parameters in neurogeriatric patients. A wearable IMU system and a validated algorithm [21] were used to acquire and process the raw sensor data. Two assessments containing two experimental walking conditions were performed within 24 h to evaluate the reliability using ICC [23] and MDC [26] for nine extracted gait parameters.

The main finding of this study is that increasing the walking distance did change the relative and absolute reliability of most parameters. Improvements were noted for all timing parameters but not for all variability and asymmetry values.

Kronenberg and colleagues [27] showed that shorter distance walks of around 15 strides are needed for reliable and valid recordings of gait variability which may explain the low relative reliability and the high MDC values in the 4 m walking condition. Increasing the number of steps allows the participant to get into a consistent walking rhythm rather than remaining only in the acceleration (at the beginning) or deceleration phase (at the end of the trial). The withdrawal of acceleration and deceleration phases during the 20 improved ICCs and MDC values (although only slightly), which further argues in favor of this hypothesis. The provided results have an important implication for clinical testing. They strongly argue in favor of analyzing longer walking distances, as this improves the relative and absolute reliability of the gait parameters investigated here. This is also supported by research conducted using pressure carpets (i.e., GaitRite or GaitMat II) showing an increase in the ICC and a decrease in the SEM values of temporal parameters with an increasing number of strides [28,29].

Gait is often affected in neurological diseases and basing treatment decisions on parameters without considering their relative and absolute reliability may be misleading. The systematic reliability assessment of the parameters allows to distinguish between values within the MDC range or values representing a real effect of a clinical intervention. In comparison to previously published research highlighting the day-to-variability of balance parameters [9], the gait parameters extracted in this study indicate higher relative reliability values (ICC) and lower absolute reliability values (MDC%) emphasizing the potential of gait as a tool for reliable clinical assessment on the ward. Hence, our results provide a benchmark when putting gait parameters into perspective and designating a change as clinically relevant.

It would be worthwhile to mention limitations of the current study and how to overcome it in practice. To begin with, the parameters highlighted here serve as a sample to illustrate the importance of systematic reliability assessment. The number of parameters that can be extracted from a quantitative gait assessment is immense and never complete. Hence it is important to also establish the reliability of other parameters of interest. The parameters extracted in this study are based on the raw data collected from the IMU positioned at the lower back. Consequently, we encourage the evaluation of parameters that are extracted from other IMU positions such as the feet or even comparing multiple analysis algorithms, to understand how the algorithm choice influences reliability. Day-to-day variabilities in sit-to-stand motions [15], balance aspects [9] and ambulation [30] have been described to occur in patients with neurological/neurodegenerative diseases (especially in PD [31]). It may thus be that gait changes between two consecutive days due to clinical reasons.

## 5. Conclusions

This study concludes that in a neurogeriatric cohort, temporal gait parameters as assessed with an IMU on the lower back, showed poor to moderate between sessions’ reliability with mean ICC and mean MDC95% values of 0.57 ± 0.18 and 52% ± 53%, respectively. Increasing the walking distance and removing the acceleration and deceleration phases should be considered to further improve relative and absolute reliabilities. As only four out of the nine computed parameters (swing time, stride time, step time and stance time) showed high relative reliability and good absolute reliability values, the selection of gait parameters for the evaluation of treatment response should be done with caution. Future reliability studies are needed that investigate additional cohorts and IMU positions on the body, to further improve our understanding about the potential of mobile health technology-derived parameters for research and clinical use.

## Figures and Tables

**Table 1:** Reliability and minimal detectable change: 4 m walking distance.

Parameter	Mean ± SD Day 1	Mean ± SD Day 2	ICC	Consistency Interpretation	SEM	MDC	MDC (%)
number of steps [n]	8 ± 2	8 ± 2	0.67	moderate	1.15	3.19	42
step time [s]	0.66 ± 0.07	0.64 ± 0.07	0.46	poor	0.05	0.14	21
stride time [s]	1.30 ± 0.13	1.26 ± 0.13	0.46	poor	0.09	0.26	21
stance time [s]	0.81 ± 0.08	0.78 ± 0.08	0.42	poor	0.06	0.18	22
swing time [s]	0.49 ± 0.06	0.48 ± 0.06	0.54	moderate	0.03	0.1	20
double limb support time [s]	0.17 ± 0.03	0.16 ± 0.02	0.4	poor	0.02	0.06	35
double limb support time variability [s]	0.05 ± 0.04	0.05 ± 0.04	0.4	poor	0.03	0.08	170
stride time variability [s]	0.08 ± 0.04	0.09 ± 0.04	0.31	poor	0.03	0.09	101
stride time asymmetry [s]	0.06 ± 0.05	0.05 ± 0.04	0.21	poor	0.04	0.11	188

ICC = intra-class correlation. MDC = minimum detectable change. MDC (%) = Minimum Detectable Change in percentage. SD = standard deviation. SEM = standard measurement error.

**Table 2:** Reliability and minimal detectable change: 20 m walking distance.

Parameter	Mean ± SD Day 1	Mean ± SD Day 2	ICC	Consistency Interpretation	SEM	MDC	MDC (%)
number of steps [n]	33 ± 5	34 ± 4	0.87	good	2.23	6.17	17
step time [s]	0.59 ± 0.06	0.58 ± 0.06	0.70	moderate	0.03	0.07	13
stride time [s]	1.17 ± 0.12	1.16 ± 0.11	0.71	moderate	0.05	0.14	12
stance time [s]	0.73 ± 0.08	0.72 ± 0.07	0.68	moderate	0.05	0.13	13
swing time [s]	0.45 ± 0.04	0.45 ± 0.05	0.82	good	0.01	0.02	14
double limb support time [s]	0.14 ± 0.01	0.14 ± 0.01	0.64	moderate	0.02	0.06	13
double limb support time variability [s]	0.01 ± 0.01	0.02 ± 0.01	0.34	poor	0.01	0.03	91
stride time variability [s]	0.04 ± 0.02	0.04 ± 0.02	0.19	poor	0.01	0.03	93
stride time asymmetry [s]	0.03 ± 0.02	0.03 ± 0.02	0.55	moderate	0.02	0.04	150

ICC = intra-class correlation. MDC = minimum detectable change. MDC (%) = Minimum Detectable Change in percentage. SD = standard deviation. SEM = standard measurement error.

**Table 3:** Reliability and minimal detectable change: 20 m walking distance (steady state).

Parameter	Mean ± SD Day 1	Mean ± SD Day 2	ICC	Consistency Interpretation	SEM	MDC	MDC (%)
number of steps [n]	30 ± 6	31 ± 6	0.87	good	2.23	6.17	21
step time [s]	0.58 ± 0.05	0.57 ± 0.05	0.65	moderate	0.03	0.08	14
stride time [s]	1.15 ± 0.10	1.15 ± 0.10	0.74	moderate	0.05	0.14	12
stance time [s]	0.71 ± 0.06	0.71 ± 0.06	0.66	moderate	0.05	0.13	13
swing time [s]	0.44 ± 0.04	0.44 ± 0.04	0.81	moderate	0.01	0.02	13
double limb support time [s]	0.14 ± 0.01	0.13 ± 0.01	0.59	moderate	0.02	0.07	15
double limb support time variability [s]	0.01 ± 0.00	0.01 ± 0.00	0.48	poor	0.01	0.01	75
stride time variability [s]	0.03 ± 0.01	0.02 ± 0.01	0.55	moderate	0.01	0.02	72
stride time asymmetry [s]	0.03 ± 0.02	0.03 ± 0.02	0.60	moderate	0.01	0.03	135

ICC = intra-class correlation. MDC = minimum detectable change. MDC (%) = Minimum Detectable Change in percentage. SD = standard deviation. SEM = standard measurement error.

## Data Availability

The data that support the findings of this study are available on request from the first author C.H.
